# Thoughts on the Pathology and Treatment of Icterus, or Jaundice

**Published:** 1828-06

**Authors:** N. Chapman


### JAUNDICE.

*Thoughts on the Pathology and Treatment of Icterus, or Jaundice.* By N. Chapman, M.D. (Condensed from the *American Journal of the Medical Sciences.*)

The disease approaches in different ways, though, as it usually occurs, it is introduced with much langour, inactivity, and depression of spirits, attended by anorexia, headache, præcordial uneasiness, considerable disorder of stomach, by flatulence and sour eructations, or nausea, or vomiting, and sometimes cramps, or colicy pains. These affections so frequently prove the precursors of the disease, that, from their existence, its invasion may with some certainty be predicted. The bowels are mostly costive, and when stools are procured they are hard, and of an ash or white colour, indicating the absence of bile. Yet in many instances there is a profusion of biliary secretion, with laxative evacuations. The urine is commonly scanty, and of a deep yellow, or brown, or saffron colour, staining linen dipped in it. There is also a very unpleasant taste of the mouth, with thirst, though the tongue is rarely changed.

As the case advances, the skin, which from the commencement is dry and sometimes distressingly itchy, becomes with the adnata variously tinged; the colour appearing first in the eyes, cheeks, neck, and chest, from which points it is progressively spread over the whole superficies of the body. But it is not uniformly diffused. I have seen it restricted to the eyes, and very often embracing only the face. As an anomaly, it is sometimes confined to a longitudinal section of the body: Behrens records a case attended by hemiplegia, where the palsied side was so exclusively icterose that only one half of the nose was coloured. Examples nearly similar are to be met with in the writings of Valsalva, Etmuller, Dupui, &c. It is most commonly of some modification of yellow, from a pale lemon to the deepest orange or saffron hue. Cases, however, are occasionally presented in which it is green, or of a darker or lurid colour, called green or black jaundice.

Not much is to be met with in the treatises on the disease, relating to this variety; and, indeed, the only precise account of it which I have seen is in a short essay by the late Dr. Baillie, so eminently distinguished by the accuracy of his observations. The substance of what he has communicated on the subject I shall notice under the several heads of my inquiry. Excepting the difference in the colour of the skin, the symptoms do not essentially vary from those I have detailed. It is said to occur more frequently in the middle and advanced periods of life than at an earlier age, though occasionally it appears in young persons, and oftener in men than women. The progress of the case is also slower, continuing a year or two, or in some instances for many years; and, though exempt from violent affections, it steadily pursues its course till the powers of the constitution are exhausted.

It has happened, however, in some instances, that the three colours have existed in different parts of the same person. Lanzoni had a patient whose face was green to the throat, while the right side of the body was black, and the left yellow. The green and black gradually became yellow, the black again resumed its colour, and finally the whole was yellow. But to return to the history of the ordinary form of the disease.

Tenderness of the epigastrium and right hypochondrium is now complained of, and fever, more or less intense, ensues; the pulse being full and strong, and sometimes hobbling, and even intermittent. Numerous are the cases, however, in which there is little or no fever, or disturbance of the circulation. The head is not unfrequently affected by pain or fulness, and the disorder of vision incident to the disease is proverbial: my allusion is more particularly to all objects being seen of a lurid or yellowish hue. Shakspeare declares

——"To the jaundiced eye
All things seem yellow."

Nor is medical authority wanting to the same effect, as that of Galen, Sydenham, Boerhaave, Van Swieten, Hoffman, &c. who assert that they have occasionally seen it. But, long and generally as the notion has been entertained, it is doubted by some whether there be any foundation for it, and certainly it is by no means a common occurrence. The fact was, I believe, first denied by Mercurialis, afterwards by Haller; and by Heberden we are told that, of all his icteric patients, he had two only who confessed its existence, and their testimony he distrusted. My own experience is decidedly against it. Whenever it takes place, the humors of the eye must be tinged, and such is a very rare event. But in other respects I have seen the vision much affected by dimness, or becoming double or inverted.

Calculi passing through the ducts of the liver are productive of spasmodic pain in various degrees of intensity, and which is often attended by vomiting so violent that nothing can be retained. The pain, however, is mostly circumscribed to the lower part of the stomach and duodenum, shooting through to the back; though such is sometimes its vehemence that there is an inability to lie down, and the sufferer, in a sitting posture, is actually drawn double. After a while relief is afforded, as is usually believed, by the escape of the calculus, which continues for days, or weeks, or months, or even for years; or, in short, till a second one gets into the same position. Even admitting this explanation as applicable to some cases with such symptoms, it surely cannot be universally received. Many are the instances in which I have known the pain removed by remedies which could have had no tendency to promote the expulsion of a calculus, and under circumstances precluding absolutely the suspicion of its existence. Most probably here it was induced by spasm of the duct or stomach, or duodenum, independently of any calculous irritation; and so analogous are the two affections, that I know hardly any criteria by which they can be discriminated. Coe, Heberden, and other writers, however, deny that icterus ever arises from spasm.

What I have hitherto said relates to the more violent and inveterate shapes of the disease. Every practitioner, however, of much experience has seen it, and with the deepest tinge of the skin, productive of no constitutional disturbance, the appetite, the pulse, the tongue, the secretory and other discharges, continuing healthy; and so little is the individual affected, that he goes about pursuing his ordinary occupations without detriment or inconvenience.

The career of the disease is very different; sometimes speedily submitting to slight remedies, or the spontaneous efforts of nature. Not a few cases of it, however, prove very indomitable, running on for a length of time, and ending in hydropic effusions, or extreme emaciation with hectic fever, or some cerebral affection, as coma, apoplexy, palsy, or convulsions.

Concerning the remote causes of jaundice, these are many and diversified, or such at least have been assigned. Commonly it is ascribed to the entrance of bile into the circulation, owing to an obstruction of the ducts of the liver from inspissated bile, or calculi, or spasmodic stricture, or thickening of the lining tissue of the tubes, or heavy hepatic congestions, or by infarctions of the duodenum, or enlarged pancreas, or by pregnancy.

Torpor or paralysis of the ducts has also been alleged to be a cause, as well as the choking up of the choledochus by worms. Examples are recorded by Beuth, Lieutaud, and Ludwig, of lumbrici being found either in the ducts or gall-bladder of icteric patients; and a preparation of the liver is in the museum of the university of Pennsylvania, illustrative of the same fact, taken from an individual who sunk under protracted sufferings from hepatitis with jaundice. But it is very often brought on by other circumstances. Certain poisons will induce it, according to Galen, who saw "a green colour to take place all over the body of one of the emperor's slaves, from the bite of a viper," which has been subsequently confirmed as to the influence of the virus of that reptile by Fontana; and I have understood the icteric aspect is a very common effect from the bite of the rattlesnake and some other venomous serpents of this country. Lanzoni tells us that "he observed an icterus occasioned by the bite of a cat, which lasted forty days;" and Van Swieten another by that of a dog. Topical yellowness is a pretty uniform effect of the sting of the bee, wasp, hornet, &c. &c. In two instances of poisoning from arsenic which came under my notice, the skin, as well as the serous exhalations in the cavities of the body, was deeply tinged with the hue.

Not unfrequently, too, the disease proceeds from mental emotions. Either vehement rage or terror has excited it, and it has resulted from petulance, grief, anxiety, and other irritating or depressing moral influences. Excessive grief, from the loss of children, caused it in two ladies whom I attended.

Concussion from the explosion of a bomb in a room, I have learned from an authentic source, once excited it almost instantaneously. Twice I have witnessed it brought on by ingesta, without spasm or other gastric uneasiness, except nausea and oppression.

Those who are most subject to the disease are the sedentary, the indolent, and studious, or whose occupations require a curvature of the body; or the dissipated, and particularly the intemperate and debauched. The hysterical and hypochondriacal are exposed to it, and all such as are harassed by the cares, disappointments, or vexations of life. Criminals are said by Haller to be peculiarly liable to it; whether from confinement or mental inquietude, does not clearly appear.

No age or sex escapes the disease. It is incident to infancy, and every subsequent stage of existence. Generally, however, it is met with in those somewhat advanced in life, and more perhaps in males than females, though the difference seems not so great as generally supposed. "Men and women," says Heberden, "are equally liable to this malady: in a continued succession of a hundred patients, I counted fifty-two males and forty-eight females."

The phenomena of jaundice are so peculiar that it can hardly be mistaken for any other disease. It is stated by Heberden "that the most distinguishing signs are a yellowness of the eyes, skin, and urine, and a want of this colour in the stools;" which symptoms are selected by Cullen in his definition of it.

Nor need much be said of the prognosis. Most cases of a recent and functional nature are sufficiently medicable, and especially so as respects those of infancy. Being, however, of long standing, and complicated with disorganizations of the liver or other important structures, or in the old and infirm in any respect, they prove generally incurable, or of very slow or doubtful amendment.

Contrary to common opinion, it is asserted by Heberden that attacks from gall-stones rarely are fatal. Nor, according to Burserius, "is there much reason to apprehend danger when occasioned by hysteria, hypochondriasis, or pregnancy, as it quickly ceases after delivery;" in which latter remark I cannot concur, having witnessed in several instances very opposite results. It has already been mentioned, on the authority of Baillie, that where the tinge is lurid or green, though the case may be protracted, recoveries seldom take place. Melæna, or the purging or vomiting of dark grumous blood, is usually a mortal occurrence, and emaciation with hectic fever, or dropsical effusion, or any heavy cerebral affection, as profound coma, apoplexy, palsy, or convulsions, imports either immediate or remote danger.

We may generally prognosticate favorably where, with improvement in the appearance of the surface, there is a subsidence of gastric disorder, more natural feces, and heavy deposits in the urine. Certain evacuations, as hemorrhage from the nose or rectum, or by perspiration, or from the kidneys or bowels, are reputed to be critical, promptly resolving the disease in some cases.

The appearances on dissection, from the history of the remote causes of the disease which has been given, must of course vary much. The liver, in certain cases, exhibits great diversity of structural derangement, while in other instances it is slightly or not at all affected. In the green jaundice, Baillie affirms "that the liver is often enlarged, hard, and tuberculated throughout its whole substance, though this morbid change of structure is sometimes confined to a single part of it, and occasionally no induration whatever is discoverable in that viscus." Gall-stones are occasionally found in its ducts as well as its bladder, varying as to number, figure, and size. "Frequently, however," says Burserius, "in icteric bodies, no morbid condition of the liver, no taint of the ductus biliferi, and no biliary calculi, are observable." This statement is substantially confirmed by Heberden. The stomach and duodenum very often betray marks of phlogosis or its consequences, by thickening or other changes of tissue. For the most part, the qualities of the bile, cystic and hepatic, are altered in colour and consistency. It has (to use the language of Good,) "been met with acid, acrid, saltish, insipid, whitish, black, green, eruginous, and versi coloured; as dense and dark as elder rob, as tenacious and limpid as the white of eggs, and as crowded and granular as the spawn of frogs."

In very violent and lingering attacks, every part of the interior of the body is tinged of the same hue as the skin. The pericardium, the heart, the abdominal viscera, the blood-vessels, the meninges and substance of the brain, the fat, the cartilages, and even the bones, have been observed in this state. Excepting the milk, all the secreted fluids, as the perspiration, the saliva, the sputum, the semen, the serous exhalations into the cavities, &c. are also found discoloured.

What is the proximate cause or true pathology of jaundice seems not determined. As before intimated, it has generally been ascribed to the absorption, more especially of cystic bile into the circulation, from some obstruction to its passage into the duodenum. That it does not depend on this clause exclusively, is shown by a case reported by Richter, where the disease occurred in an individual who, after death, was found destitute of a gall-bladder; and scarcely less conclusive is the result of an experiment by Portal, in which he tied the cystic duct of an animal without producing jaundice. I have long entertained doubts whether it could be assigned, under any circumstances, to either species of bile. It appears to me that, were it owing to this cause, the disease might at any time occur, or whenever there is bile exposed to the action of the lacteals or other absorbents, as when accumulated in the stomach and small intestines; that, though the stools usually indicate a want of bile, this is not uniformly so; that in many instances of the disease no obstruction existed, on a post-mortem examination in the ducts of the liver, or any other derangement, to account for it; that the peculiar bitter taste of bile is not discernible, and that, were it floating in the circulation, it should tinge the whole mass of blood, which it does not, the yellowness being apparent only in the serum on the separation of the constituents of the blood.

To these arguments it may be added, that in this disease there is very rarely any genuine bile formed. The fluid, discoverable on dissection, as well hepatic as cystic, has scarcely any such properties as to taste, colour, or consistency. It is a peculiar one, the result of a morbid secretory action, caused by the condition in which the liver is placed. Even admitting that absorption takes place, as is contended for, the tinge imparted to the skin should then be of a correspondent colour, and not of the various shades of yellow, which happens. But such a process no more goes on in this case than in retention of urine. The bile must be taken up from the pori biliarii or hepatic or cystic ducts, or gall-bladder, which is contrary to all analogy and fact. Does it ever happen in relation to the urine under such circumstances? Conceding that genuine urine has been discharged from the stomach and other remote parts of the body, of which some instances are reported, they supply no evidence of its absorption. These parts have assumed a vicarious office, as we have frequent occasion to witness with regard to menstruation.

Embarrassed by these difficulties, some of the advocates of the hypothesis which ascribes jaundice to bile have so far changed their ground, as to allege the greater probability of its being introduced into the blood by *regurgitation,* or lacteal absorption. As to the first of these suppositions, its absurdity is so obvious that it has received a very slender support; and, in relation to the second, experience refutes it. By Powell, a recent and authoritative writer on the subject, we are informed that the disease "never accompanies those cases of immense secretion of bile which are called cholera; at least I have never seen it in very violent ones; nor do I know any author who mentions it, even as an accidental symptom, and, if it had happened, it could not possibly have been overlooked. Bianchi," continues he, "gives a case where this circumstance was more narrowly investigated, for he examined the lacteals of a man who had died of cholera, and found that their contents were not in the least tinged by bile." Let me repeat that there is no absorption of bile. The secretory function of the liver in jaundice, so far as concerns the healthy exercise of it, is suspended, as in bilious or malignant fever; and the earliest manifestation of recovery is the restoration of its office.

Denying, therefore, altogether these several hypotheses, I am led to believe that in some undefined irritation of the chylopoietic viscera, arising from any of the remote causes formerly enumerated, with which the capillaries sympathise, the serum of these vessels undergoes a morbid change, as in yellow fever, and in certain cases of poisoning, &c. The same discoloration which occurs in a part from a bruise happens in all these instances, and is equally referrible to the torpor or impaired vitality of the extreme vessels. The capillaries being in this languid state, no matter how it may be induced, are disposed to secrete a fluid of some shade of yellow or its combinations, thus tinting the surface, &c. We meet with it in the cachexies, &c. It sometimes happens in the diseases of the spleen. I have seen it as an attendant on gastric or enteritic epilepsy, and very conspicuously in the chlorotic affections, where the complexion exhibits all the gradations of hues from the icterose to the green and livid. As strongly is it illustrated in the cutaneous efflorescences, especially when of a weak or typhoid character. Cases of rubeola, scarlatina, and erysipelas, have repeatedly come under my care, followed by this sallowness. But the most striking proof is offered in some of the early eruptions of infancy. These, at first florid, on the weakening of the vessels gradually assume the sallow hue. The act of death is often productive of the same effect. It is indeed a very common occurrence, however pallid before, for the corpse to become all over yellow. But it may be asked, does not jaundice also tinge the internal structure of the body? Be it admitted, and what can be deduced from it? Does it not often occur where it cannot possibly be imputed to bile? Moreover, are not the capillaries distributed through every part, the most minute and obscure recesses of the animal machine, and, as the cutaneous, so are the rest affected?

Considerations such as I have presented seem to demonstrate that jaundice is not owing to the absorption of bile, and we are left, as I conceive, to seek for the cause of it in that state of the extreme vessels which has been described. The parent irritation of the chylopoietic viscera, which is very commonly, I suspect, in the mucous tissue of the stomach or duodenum, and not the liver, is extended to the capillaries, and, in consequence of the condition thus induced, changes are wrought in the serous fluid circulating through them, characteristic of the icteric affections. Complexional hues being bestowed in this manner in various other cases, why should it not also happen in jaundice? That the closest similarity exists in the whole of these affections is undeniable, and it remains for those who are opposed to the views I have endeavoured to sustain, to point out the peculiar and distinctive character of jaundice.

It is sufficiently apparent, from the preceding account of jaundice, that the prospect of effecting a cure cannot be the same in the different cases; and, to be appropriate, our remedies must be varied according to circumstances. The remote and not the proximate cause of it we are called upon to remove in many instances, and, from the diversity of circumstances by which it is induced, there can be no uniform or consistent plan of management adopted. Nor is this the only difficulty to be encountered. Cases dependent on the most opposite conditions, and exacting quite a different curative process, are so obscurely designated as to elude all the powers of discrimination. It is on these accounts that our practice is nearly always tentative, and, I fear, too often degenerates even into absolute empiricism.

In the ordinary forms of the disease, where little fever or local pain exists, we commence the treatment with evacuations of the primæ viæ, and emetics are especially useful.

Cathartics are also important, so much so, indeed, as to constitute a very essential, and not unfrequently the only remedies. Beginning with active purging by calomel alone, or with its ordinary adjuncts, the bowels are subsequently to be kept in a soluble state by magnesia, the neutral salts, or other gentle laxatives.

These two classes of remedies operate probably on nearly the same principle, removing causes of gastric or intestinal irritation, and by exciting the liver sympathetically, through the strong impression made on the alimentary canal. In proof of the beneficial tendency of purging in particular, it may be repeated that jaundice is often cured by the spontaneous occurrence of diarrhœa.

The case I have presented, one of the simplest of the disease, is comparatively easy of cure. But it wears a more complicated aspect, and demands a different course of proceeding. Coming on with fever, or a strong full pulse, and topical uneasiness, venesection must be practised, and to some extent, to remove such a condition of the system. Greatly may it be aided by topical bleeding, which is too much neglected. The stomach, the duodenum, and the liver, are here all involved in irritation, congestion, or phlogosis. By a detraction of a few ounces of blood from the epigastric or right hypochondriac region, according to the indication, I have seen signal advantage to result, and, where the means of local bleeding cannot be had, blistering may be substituted. Not less are these means required in those distractions of the head which sometimes prevail for days, and relief not being afforded, prove one of the most afflictive attendants on the case.

What, however, calls for the most vigorous proceedings, is a relentless obstruction of the biliary ducts, from a calculus or the spasm of these or of the stomach, &c. inducing the intense suffering formerly noticed.

The indication here is twofold, to induce relaxation of the duct, &c. so as to overcome the impediment, and to obviate inflammation, which would follow were obstruction or the spasm to continue. To meet these views, we resort to copious venesection, sometimes even *ad deliquium,* to the warm bath, topical fomentations, and to bleeding by cups or leeches, to vesication, to anodyne enemata, and emetics. With regard to the latter, their use must necessarily be precarious and doubtful, to the expulsion of calculi.

These are found of various sizes, from that of a small granule to a hen's egg. It is obvious that, when the stone is so great as not to pass through the duct, vomiting must be mischievous; and it is utterly impossible to ascertain its dimensions. All that we can do is to be governed by the symptoms. Much pain, fever, and general excitement existing, emetics are to be avoided.

Being relieved of this urgent affection, the case next calls for the means which are supposed by many to have the power of dissolving or otherwise destroying biliary concretions. But I believe they operate on no such principle, and it is not easy to explain their efficacy. Be their modus operandi as it may, experience has taught that some of them are useful. Ether and the spirit of turpentine mixed had once a high reputation under such circumstances, though now little or not at all employed. Ever having done good, I suspect it was by their carminative or antispasmodic powers, and not from any efficacy as a solvent or deobstruent. Combinations of the alkalies, in the mild as well as the caustic state, have also long claimed, and perhaps justly, much attention: they are variously administered, though the ordinary shape is that of Castile soap. Equal portions of it, rhubarb, and aloes, with or without calomel, I have often known to be effectual. The common potash mixture, prepared agreeably to the annexed formula, is likewise serviceable in a table-spoonful dose occasionally.* But incomparably the best preparation which I have ever tried is the following popular nostrum.†

Many articles which must act in a very different mode from those alkaline remedies, are entitled to confidence. The acids, mineral as well as vegetable, are among these, and especially the nitric, which is by some highly estimated. Cider or even lemonade, I have known to be serviceable.

As a general rule, however, the treatment consists principally in keeping up a pretty constant impression on the primæ viæ by purgatives, with which view the pill or the syrup of the butter-nut is well entitled to notice.‡

Next, where the case does not give way, an alterative course of mercury should be resorted to; and this failing, the nitro-muriatic acid exhibited internally, as well as applied externally by frictions or as a bath, may be tried. It is at this juncture that the Taraxacum, the dandelion of our fields, has been much trusted to by some practitioners.

Directly the reverse, however, of this plan of management is recommended, consisting alone in the narcotics, as cicuta, hyosciamus, belladonna, the prussic acid, &c. To alleviate spasmodic pain, or to quiet irritation, I give opiates, and for no other purpose resort to narcotics, believing that these are the only purposes which they are capable of fulfilling at this stage of the disease.

Of the icteric cases dependent on disorganised conditions of the liver, I shall here say nothing. Being merely effects

* ℞. Potassæ Carbonas, Gum. Arab. āā ʒj.; Tinct. Theb. gtt. xxx.; Ol. Menth. gtt. x.; Aq. font. ℥iv.

† ℞. Carbonas Potassæ ℥j.; Sapon. Hispan., Gum. Arab. āā ℥ss.; Alcohol dilut. ℔j.—To be frequently stirred, so that the ingredients may be well mixed and dissolved, which will require several days. The dose is half a wine-glassful, to be taken for three successive mornings fasting; and, if not relieved, omit it for one day, and then recur to the same mode.

‡ The Juglans Cathartica of Michaux.

of another disease, the cure can only be accomplished by its removal; to point out the treatment of which is alien to my present design. Little more, therefore, remains to suggest as to the management of icterus, than that advantage is sometimes derived from the use of those mineral waters which are found appropriate to the other hepatic derangements. Even a journey to the Springs, and particularly when taken on horseback, is occasionally useful, not probably, as is generally imagined, so much by dislodging calculi, as invigorating the primæ viæ or the secretory action of the liver. It is on the same principle that electricity and galvanism have been applied to the cure of these cases.

Too much, however, is usually done in the treatment of this disease. As long as the sallowness of the surface endures, it is thought by many practitioners, and always by the patient, that there is occasion for the continuance of active remedies. But this colour of the skin is merely an effect, over which we have little control; when unconnected with constitutional disturbance may be disregarded, or left to the natural or recuperative powers to remove. From an opposite course, harassing the system, and especially the primæ viæ, by such measures, I have certainly seen a very serious derangement of health induced, and in some instances permanently entailed.

Convalescence is more effectually promoted by a duly regulated regimen, very similar to that in dyspepsia, and, should the stomach suffer, by the moderate use of the vegetable bitters or mineral tonics, and particularly the mildest of the martial preparations, so united with rhubarb as to obviate costiveness. An oppression in the region of the duodenum is not unfrequently felt an hour or more after a meal, owing, perhaps, to an accumulation of food from torpor of that intestine; to relieve which nothing answers so well as a couple of ounces of the infusion of senna and gentian, in the proportion of half an ounce of the former to a drachm of the latter, in a pint of water.

An icterose predisposition being established, and which is generally laid by an attack of any severity, speedy relapses, or more remote recurrences of the disease, are very apt to take place. To guard against these is an important consideration, as the constitution thus repeatedly assailed becomes disordered, and ultimately a train of morbid consequences arises of the most fatal import. It will be well studiously to avoid all the exciting causes, and which are chiefly embraced in want of attention to the bowels, inappropriate clothing, indiscretions in diet, exposure to the fluctuations of weather, inordinate exercises or the reverse, habits of indolence, the indulgence of intemperate passions, or the cherishing of anxieties or cankering cares. This is a prophylactic precept, which should always be inculcated and strictly observed.

